# Supplementary material for: Transcription Factor Action Orchestrates the Complex Expression Pattern of CRABS CLAW in Arabidopsis
Source: Genes (Basel). 2021 Oct 21;12(11):1663. doi: 10.3390/genes12111663 (PMC8653963; doi:10.3390/genes12111663)
Supplement: Supplementary file 1 [file genes-12-01663-s001.zip › genes-1390869-supplementary.pdf]

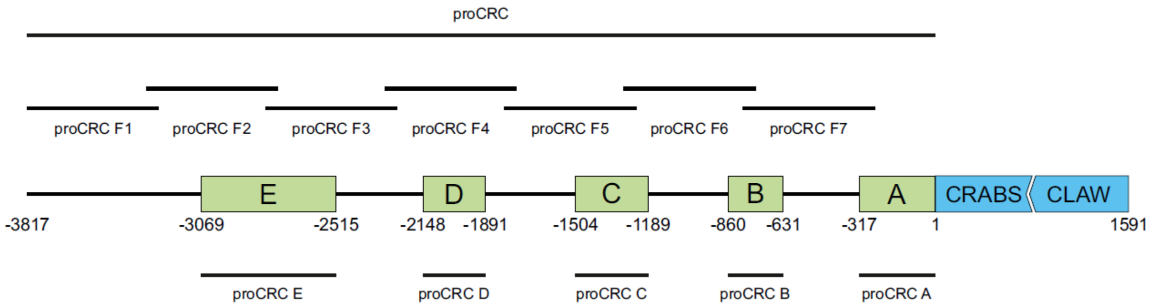

Figure S1: Schematic representation of the *CRC* promoter fragments used for the Y1H screen. The CRC promoter was used in full length, and divided into shorter fragments (F1-F7) with usually around 550 bp length. In addition, the conserved promoter regions A-E were used as bait fragments in the Y1H.

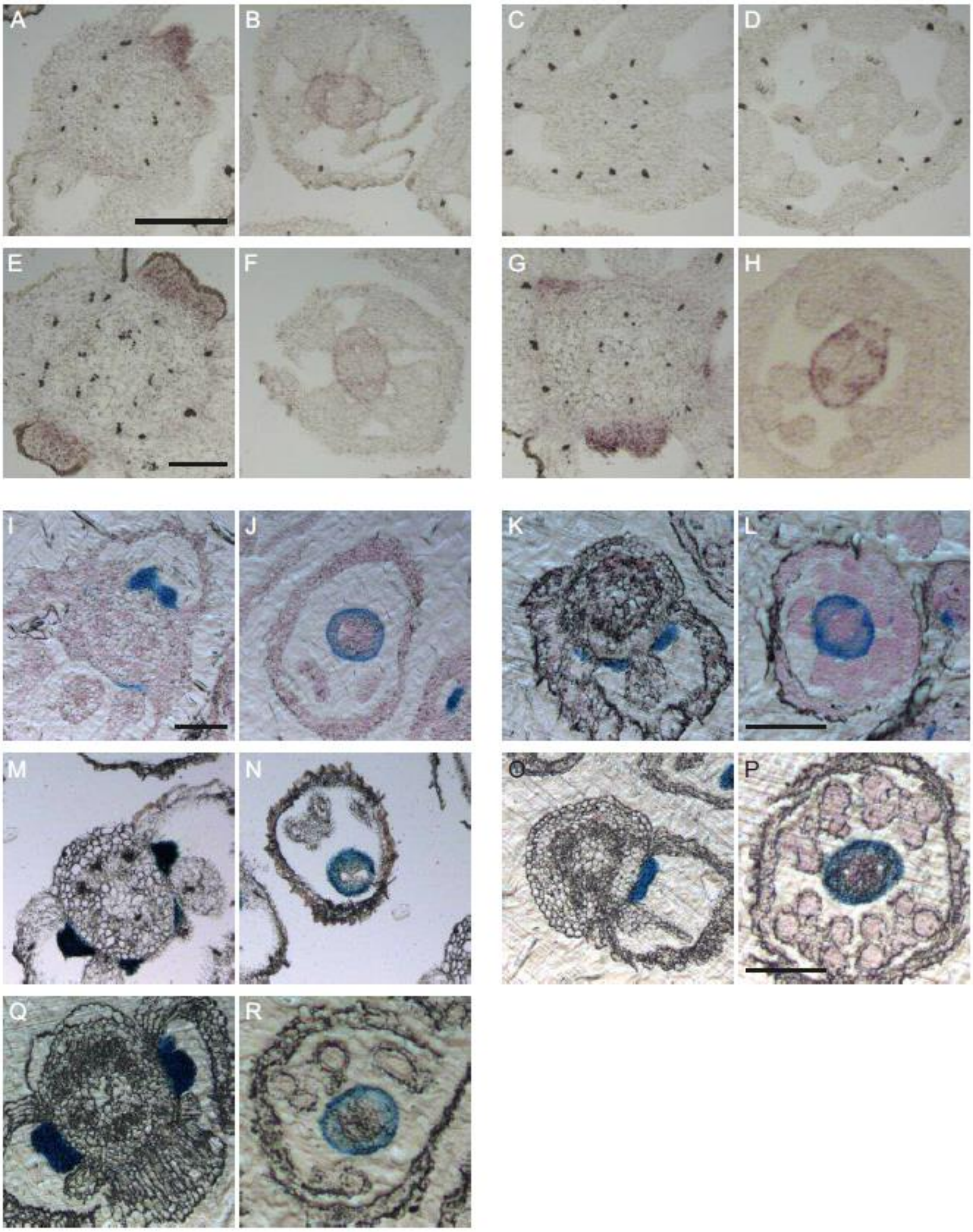

Figure S2: Spatial analysis of *CRC* expression with RNA *in situ* hybridization and GUS staining. A-H: In situ hybridization using a *CRC* antisense probe of *A. thaliana* Col-0 wild type (A, B), *crc-8* (C, D), *cal* (E, F), and *hbb* (G, H) buds. A, C, E, and G show CRC expression in the nectaries, and B, D, F, and H show CRC expression in approximately stage 9 gynoecia. Scale bars represent 100 µm (identical in A-H, except for E). GUS stainings of *A. thaliana* Col-0 *proCRC:GUS* (I, J). For K-R the *proCRC:GUS* reporter line was crossed with *agf2* (K, L), *athb16* (M, N), *bbx19* (O, P), and *ino* (Q, R). I, K, M, O, and Q show *CRC* expression in the nectaries, and J, L, N, P, and R show *CRC* expression in approximately stage 9 gynoecia. Scale bars represent 100 µm (identical in I-R, except for L and P).

The RNA in situ protocol is based on Brewer et. al (2006) and was modified by Andrea Gómez Felipe. Inflorescences of the respective *A. thaliana* plants were collected, fixated, embedded, and sectioned as described in Brewer et al. (2006). For probe synthesis and labeling, the DIG RNA Labeling Kit (SP6/T7) (Roche Diagnostics GmbH, Mannheim, Germany) was used. Paraplast was removed by dipping the slides in 100 % Rotihistol (Carl Roth GmbH + Co. KG, Karlsruhe, Germany), followed by a decreasing ethanol series to fully rehydrate the sections. An acid hydrolysis with 0.2 M HCl for 20 min at room temperature was performed and the sections were washed with DEPC treated H_2O and 2x SSC. This was followed by Proteinase K treatment and post-fixation according to Brewer et al. (2006). The pre-hybridization step was omitted but the hybridization step was carried out at 50 °C for one day as described. Following hybridization, the slides were washed twice in 0.2x SSC for 60 min at 55 °C and for 5 min in TBS buffer (100 mM Tris-HCl pH 7.5 and 150 mM NaCl) at room temperature. Labelled probes were detected using the DIG Nucleic Acid Detection Kit (Roche Diagnostics GmbH). For this, a 0.5 % blocking solution in TBS was made and the slides were incubated for 60 min at room temperature. The slides were washed with TBNT buffer (1% BSA, 0.3% (v/v) Triton X-100 in 1x TBS) for 30 min at room temperature and incubated with the in TBNT 1:2000 diluted DIG antibody at room temperature for 2 hours. Slides were washed three times in fresh TBNT buffer for each 20 min at room temperature. Afterwards, slides were washed with detection buffer (100 mM Tris (pH 9.5), 100 mM NaCl, and 50 mM $MgCl_2$) for 5 min at room temperature. The staining solution NBT/BCIP was diluted in detection buffer according to the kit's manual and 200 µl were placed on each slide, which were then incubated at room temperature. After two days the staining reaction was stopped by washing the slides in TE buffer (10 mM Tris pH 7.5, 1 mM EDTA pH 8.0). Slides were dried and mounted with glycerine gelatine.

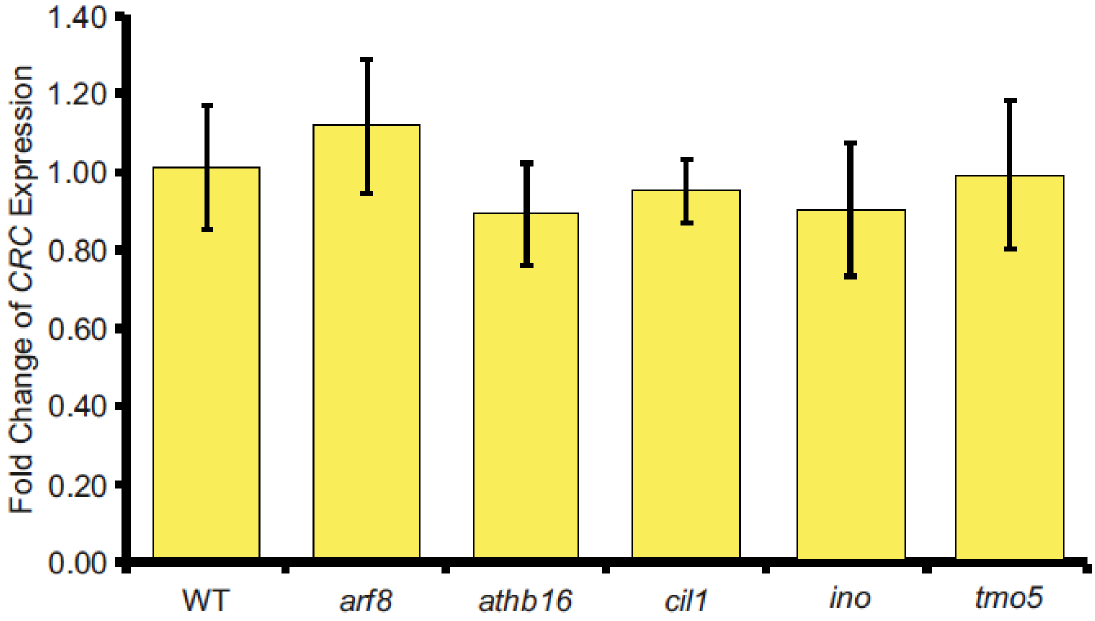

Figure S3: qRT-PCR analysis of the different mutant lines in regard of their *CRC* expression. The y-axis shows the relative expression of *CRC* as fold change with *ACTIN2* used as housekeeping gene for normalization. The expression of *CRC* is referenced against the *CRC* expression in buds of Col-0 wild type plants. Buds of the respective plants (samples were taken from four biological replicates) were harvested in liquid nitrogen and stored at -80 °C upon use. Using a mortar, the tissue was ground to a fine powder and 100 mg of the powder was added to the lysis buffer (RA1, NucleoSpin RNA Plant kit, Macherey-Nagel, Düren, Germany). Total RNA extraction was performed according to the manufacturer's instructions, including an on-column DNase treatment. The RNA concentration was determined spectrophotometrically using a Nanodrop 2000c (Thermo Fisher Scientific/Peqlab) and RNA integrity was checked via agarose gel electrophoresis. Samples that showed smear or degradation of the rRNA bands were discarded. For cDNA transcription, 1000 ng of total RNA were by using the RevertAid H Minus Reverse Transcriptase Kit (Thermo Fisher Scientific Inc., Schwerte, Germany) using random hexamer primers according to the manufacturer's instructions. Primers of the two tested genes *CRC* (NM_105585.3) and *ACTIN2* (NM_001338359.1) were used as described in Gross et al. (2018) (see Suppl. Table 2 for sequences). The exon spanning primers (*ACT2*: Fw in Exon 1, Rv in Exon 2; *CRC*: Fw in Exon 2, Rv overlapping Exon 3 and 4) were ordered from Eurofins Genomics (Ebersberg, Germany) without any modifications. For CRC an amplicon of 181 bp was expected and 93 bp for ACT2. The cDNA was diluted in a dilution series (1:10, 1:100; 1:1000; 1:10000) and used as a template for qRT-PCR to determine the amplification efficiencies of the respective primer pairs. A standard curve was calculated in Excel 2019 (Microsoft, Redmont, USA) and the slope (*CRC*: - 3.01, R^2: 0.99; *ACT2*: -3.03, R^2: 1) was used to determine the respective amplification efficiency (*CRC*: 2.1; *ACT2*: 2.1). The Luna® Universal qPCR Master Mix (NEB, Frankfurt am Main, Germany) was used for qRT-PCR according to the manufacturer's instructions and 1 µl of a 1:50 cDNA dilution (20 ng) was added as template. Lightcycler 480 II (Roche Diagnostics Deutschland GmbH,

Mannheim, Germany) was used for qRT-PCR with 95 °C for 60 s, 95 °C for 10 s, 60 °C for 10 s, 72 °C for 10 s and 45 cycles, followed by a melting curve analysis, in which only the expected fragments were detected. Two technical replicates were performed. The obtained data was analyzed using the Pfaffl method (Pfaffl, 2001).

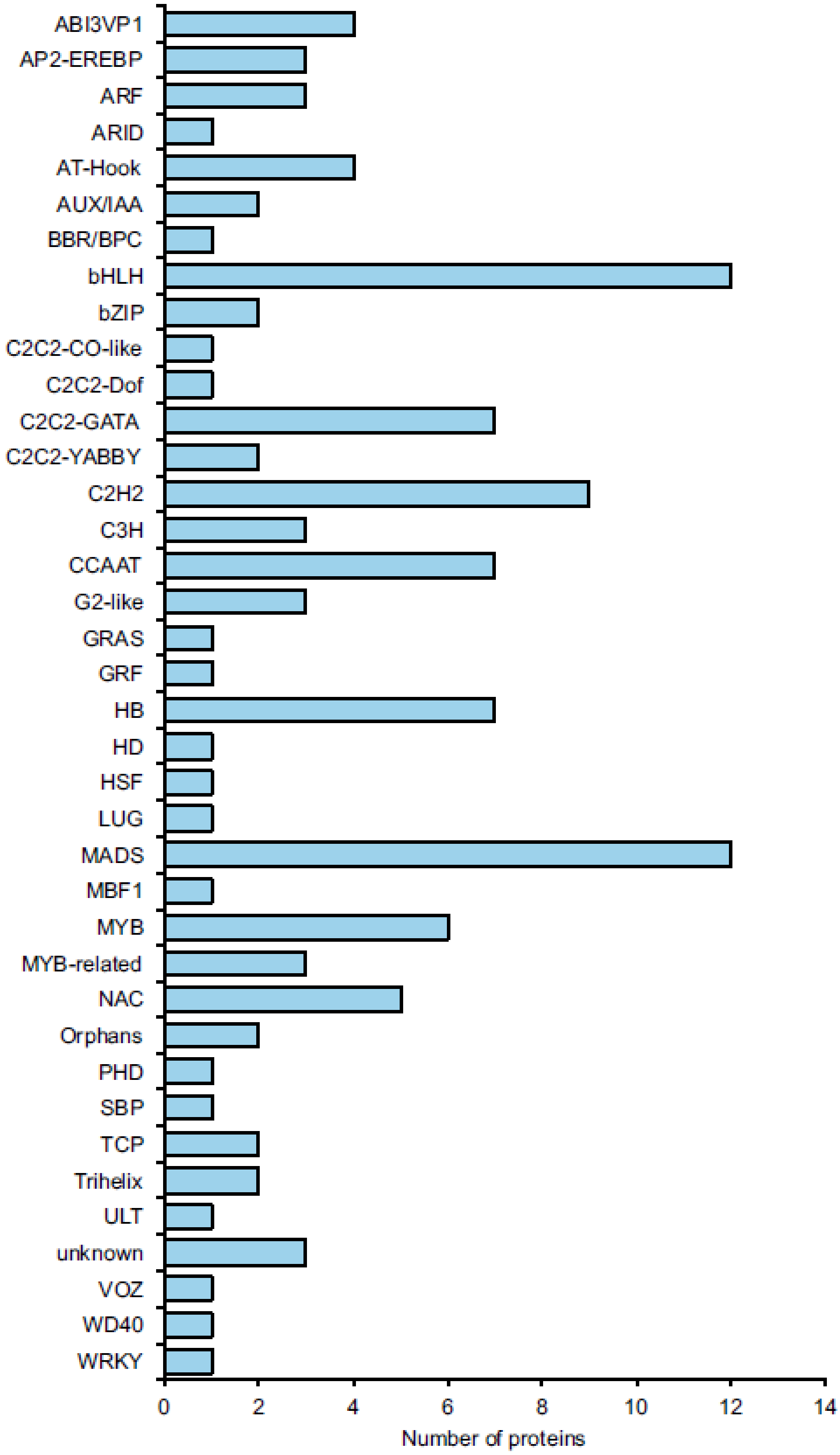

Figure S4: Protein families of the identified transcription factors. Classification into families was based on TAIR and PlnTFDB. Shown are the family name (y-axis) and the number

of identified family members (x-axis)

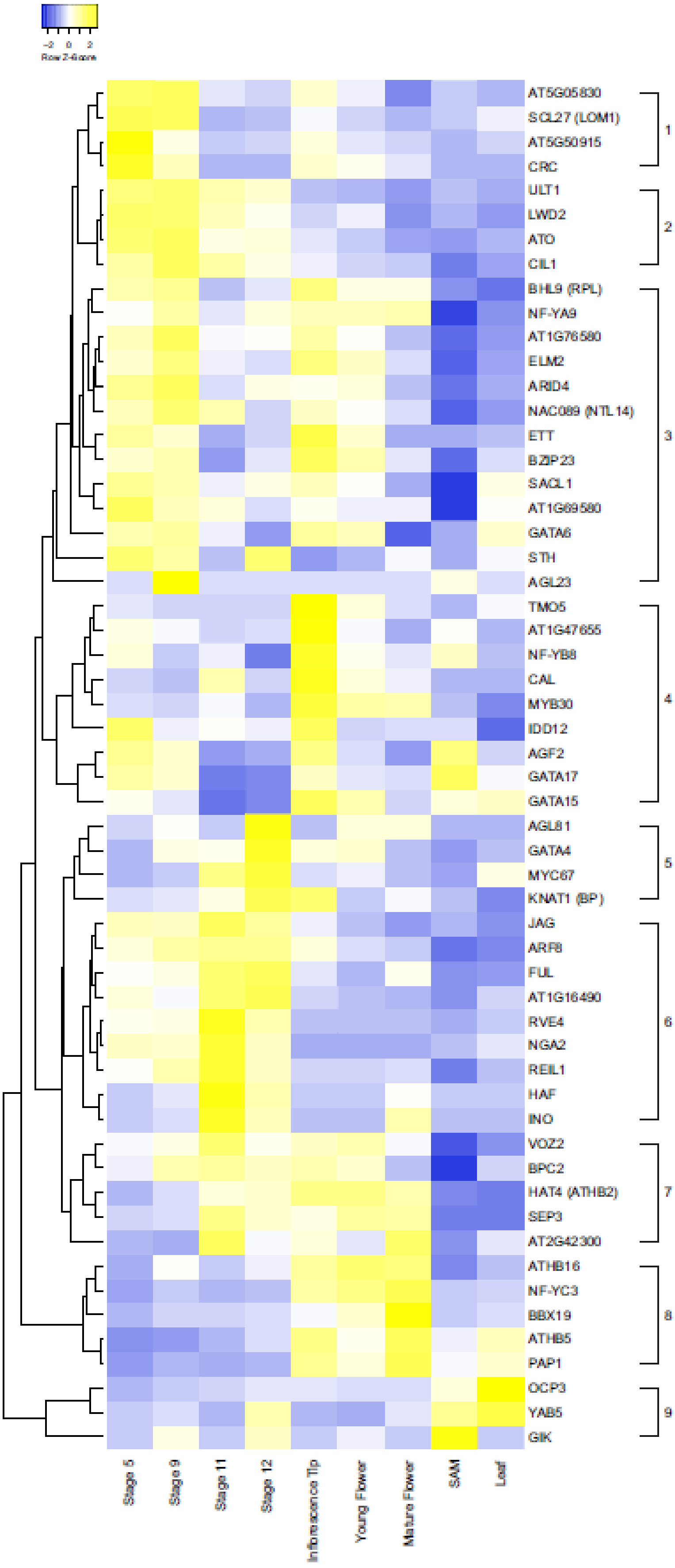

Figure S5: Heatmap of *CRC* and co-expressed putative regulators during four carpel

developmental stages, inflorescences, young flowers, mature flowers, shoot apical meristem
(SAM), and leaves. The rows are not correlated to each other. Color intensity represents zscore.

Table S1：SALK mutant lines

Gene Name	Locus ID	Accession ID	Salk/Sail Line
IDD12	AT4G02670	N2104223	SALK_208784C
AGF2	AT3G55560	N526607	SALK_026607
NF-YA9	AT3G20910	N671031	SALK_002235C
TMO5	AT3G25710	N68622	SALK_013517C
FUL	AT5G60910	N563068	SALK_063068
HAT4	AT4G16780	N506502	SALK_006502
ATHB16	AT4G40060	N524956	SALK_024956
RVE4	AT5G02840	N618847	SALK_118847
BBX19	AT4G38960	N657986	SALK_087493C
CIL1	AT1G68920	N661158	SALK_135188C
ETT	AT2G33860	N24604	SALK_005658
ARF8	AT5G37020	N527141	SALK_027141
JAG	AT1G68480	N6945	
YAB5	AT2G26580	N668134	SALK_041504C
GIK	AT2G35270	N669949	SALK_094394C
INO	AT1G23420	N673265	SALK_116219C
ULT1	AT4G28190	N675794	SALK_074642C
NGA2	AT3G61970	N677082	SALK_137356C

Table S2: Primer sequences

	Primer	Sequence (5' -> 3')	Published
	pCRC Fw Hind	ATTAAAGCTTCCGATCGAGGTTAGGAAA	
	pCRC Rv Kpn	TCTAGGTACCGGTCTTTAGCGAATGGATTG	
	pAbAi Seq	GTTCCTTATATGTAGCTTTCGACAT	
	pCRC Seq 2	TTCTAACTTTGAGAGCAAACTTC	
	pCRC Seq 3	ATGTGTCTGAAGAAGATTCATTG	
	pCRC Seq 4	AAGATTTTGCAGAGGGAGG	
	pCRC Seq 5	GTTGTACCACTAAAACACC	
	SDM pCRC Fw	TATATATATATGTCATCGTCTCACTATGATTGTTC	
	SDM pCRC Rv	TAGAGAGAGAGAGAGAGAGCGATACAG	
	A pCRC Fw BsaI	AACAGGTCTCAACCTCCGATCGAGGTTAGGAAA	
	A pCRC Rv BsaI	AACAGGTCTCATGTTGGTCTTTAGCGAATGGATTG	
	MAS:Basta R Seq	TTACGTCACGTCTTGCGCA	
	pCRC Frag 1 Rv	ACATCTCGAGTAAACTTCCCTGAGCGATCT	
	pCRC Frag 2 Fw H	ACATAAGCTTATTTCTTGTTTTTCTAATTAGGG	
	pCRC Frag 2 Rv	ACATCTCGAGGAGATGGGACGATTGCC	
proCRC cloning	pCRC Frag 3 Fw H	ACATAAGCTTTAAACCCTAATTTCTTATTAGC	
	pCRC Frag 3 Rv	ACATCTCGAGGAAGAAAACATGAATAACTAACTA	
	pCRC Frag 4 Fw H	ACATAAGCTTGTTGATTTTGGAATTAGACTACC	
	pCRC Frag 4 Rv	ACATCTCGAGCGCTTTATTGTTGAAATTTGAGA	
	pCRC Frag 5 Fw	ACATAAGCTTCATCTTCTCTATAATTAGTATGC	
	pCRC Frag 5 Rv	ACATCTCGAGGGTCCTTTCCTGATCTTTTG	
	pCRC Frag 6 Fw	ACATAAGCTTCTCAGTTTTGCAGTGAAATC	
	pCRC Frag 6 Rv	ACATCTCGAGCTGCAAAATCTTGCAGACG	
	pCRC Frag 7 Fw	ACATAAGCTTAGTGACATTTAGGGTCTTG	
	pCRC Frag 7 Rv	ACATCTCGAGAACAACATTTAATATCATCTTATC	
	pCRC Frag 8 Fw	ACATAAGCTTCTCGTGTCTACACCAGAAT	
	pCRC E Fw Hind	ACATAAGCTTTCATTCAATAATTAAGTCGACTAAGC	
	pCRC E Rv Xho	ACATCTCGAGATCTCATCATTGGCATTAAGAGAC	
	pCRC C Fw Hind	ACATAAGCTTAATGTATGTATAGTTGGATGTGTC	
	pCRC C Rv Xho	ACATCTCGAGATGGTGTGAATATGATTACATTTAT	
	pCRC A Fw Hind	ACATAAGCTTCTCGTGTCTACACCAGAAG	
Y1H library sequencing	AD Rv Mitsuda	CGTTTTAAAACCTAAGAGTCAC	Mitsuda et al. (2010)
	AD Fw Mitsuda	ATTCGATGATGAAGATACCCC	Mitsuda et al. (2010)
	LBb1.3 tDNA	ATTTTGCCGATTTCGGAAC	
	IDD12 LP	GGTCGCATGTTCTTGTTTTTG	
	IDD12 RP	TCCTATGTCGGCGTACGATAC	
	MYB30 LP	TCCTTGTTGTGACAAAGGAGG	
	MYB30 RP	ATGATCAGGTGAAACACCAGC	
	STH LP	AGCCCAGAAAGAGAACTGAGG	
	STH RP	GCCTTTTGTTTCTTTCCCTTG	
	AGF2 LP	CAGGAAGAGAATCCGAAAACC	
	AGF2 RP	CACCACTCTCTTTGCATATAATCC	
	PAP1 LP	AATGCCTCACCCATTTCTTG	
	PAP1 RP	TTCTTGGACAACCTTCCATTG	
	NF-YA9 LP	TGGCTATTGTTGTTGTCATGC	
	NF-YA9 RP	AACTGTGAACGCATCACACTG	
	NF-YB8 LP	CTAAGCCCATTGATATCGTCG	
	NF-YB8 RP	CTGCACGTATTCTCTCTTCCG	
	TMO5 LP	TTTGCCTCTTAATACCCCCTG	
	TMO5 RP	TGAGTGCACAAGAAGTCATGG	
	FUL LP	AATTGTCCTTCTTGCTGACCC	
	FUL RP	CGATCGAGAAGTTGAGTTTGG	
	HAT4 LP	AGACCCAGATCGTCTTTCTCG	
	HAT4 RP	AAAGTAAAACTCATGCGGTCG	
	ATHB5 LP	AGAGGAAAGTGAAGCTGGCTC	
	ATHB5 RP	TGAGTAATGCATTTTCCGACC	
	ATHB16 LP	CACACATTGAATCTGAGCTGC	
	ATHB16 RP	ATTGTCTCTCGGAAAAGCTCC	
	RVE4 LP	CTGCAGAGGAAGGTCATGAAC	
	RVE4 RP	CCTGTTAACCCTAATCTCGCC	
	BBX19 LP	ATGGGGCCTTTGCATATTAAG	
	BBX19 RP	AATGAACTTCCCACACTCGTG	
	CIL1 LP	TTTCCGTCGTAACAACGAATC	
	CIL1 RP	CTAGTACCGGGTTGCAACAAG	
	AGL83 LP	GATCTGTGCATCGGAGAGAAG	

Salk line genotyping	AGL83 RP	GTCTTGTAGCGCAAACTACGC	
	AGL23 LP	TCCTTTAACCAATCATTGGTACC	
	AGL23 RP	ACCACCACTAACAGTTGCTGG	
	AGL81 LP	TTACATTTCCGCCCTAACTCC	
	AGL81 RP	TTGCTTTCTTCTCCAAGTTCG	
	VOZ2 LP	CTCTTGTCGTCTGCTGTCTCC	
	VOZ2 RP	AAGTGTGCACTATGGGATTGC	
	Haf-x8	CATCAAGCATCACTGCCATT	
	DS3-2	CGATTACCGTATTTATCCCGTTC	
	BEE1 Fw	CCCGGAAACTCTCCAGACAGTAGTAACAA	
	BEE1 Rv	CCTTATAACATCCGGGCACCATATCTTGCA	
	BEE3 Fw	CTCTACCTCTTCTGCTCAAGTTTCCATAAA	
	BEE3 Rv	AATCATAGCAAACATCACCAGTCTTACGAG	
	ETT LP	TGCATAGATGTCCCTTCCTTG	
	ETT RP	GGATGAGATTTAAAGCGAGGG	
	ARF8 LP	AACCGTTGTCACTTCCACAAG	
	ARF8 RP	CTTTGGGTCAAAAAGAAAGGG	
	RPL LP	TTGGAACCAAGTTCAAACTCG	
	RPL RP	ATGTTCACAGTTTTTGGTCGG	
	SEU LP	AACACAACCTGCCAACATTTC	
	SEU RP	CTTGTGGAATGAAATTTTGCG	
	KNAT1 LP	GAGTTTCCAGCTTCTGACACG	
	KNAT1 RP	TCCAATCAACAAACAATGCAG	
	LUG LP	TTAACATGGAGACGCAAAACC	
	LUG RP	TGGGTTGAAGATTCTGAATGC	
	YAB5 LP	GTGTAGGTGAATGTCCCATGC	
	YAB5 RP	GTACGCAGAAGGTACTCGCTG	
	GIK LP	TCACCAACTACGTTACCTCCG	
	GIK RP	AATCCCATTTTAGTCCGTGTTG	
	INO LP	AAGCTCTGCCTTTCCTTTGTC	
	INO RP	TGTCATTTTCCAAAGCAAACC	
	ULT1 LP	TTTGACAATGGAACCTTTTCG	
	ULT1 RP	TCTTCTCTTCTCCCGAAAAGC	
	NGA2 LP	GTCGTCAGGTCCTAACGTTTC	
	NGA2 RP	ATGGTGGTGGATGAGATTGAC	
in situ probe	AtCRC_SE_T7_fw	TAATACGACTCACTATAGGGAGACTCTCGTTTCTCACCACAACTC	
	AtCRC_AS_T7_rv	GCTTCTTCTCAGGAGGTTTGACTCTCCCTATAGTGAGTCGTATTA	
qRT PCR	AtCRC_qPCR_fw	CTCTCGTTTCTCACCACAACTC	
	AtCRC_qPCR_rv	GCTTCTTCTCAGGAGGTTTGAC	
	RTq-At-Actin_Fw	AGTGGTCGTACAACCGGTATTGT	
	RTq-At-Actin_Re	GATGGCATGGAGGAAGAGAGAAAC	

Table S3: Y1H libraries

Locus	Name	Family	in PP	motif in PP	predicted binding site	Inferred or te
AT1G10120	CIB4	bHLH	Yes	Yes	Yes	Both
AT1G23420	INO	C2C2-YABBY	Yes	Yes	Yes	Tested
AT1G24260	SEP3	MADS	Yes	Yes	Yes	Tested
AT1G25330	HAF	bHLH	Yes	Yes	Yes	Inferred
AT1G26310	CAL	MADS	Yes	Yes	Yes	Inferred
AT1G47655		C2C2-Dof	Yes	Yes	Yes	Tested
AT1G50680		AP2-EREBP	Yes	Yes	Yes	Inferred
AT1G54160	NF-YA5	CCAAT	Yes	Yes	Yes	Tested
AT1G54830	NF-YC3	CCAAT	Yes	Yes	Yes	Tested
AT1G59750	ARF1	ARF	Yes	Yes	Yes	Tested
AT1G67260	TCP1	TCP	Yes	Yes	Yes	Both
AT1G68670	HHO2	G2-like	Yes	Yes	Yes	Tested
AT1G68920	CIL1	bHLH	Yes	Yes	Yes	Both
AT1G76420	CUC3	NAC	Yes	Yes	Yes	Both
AT1G76580		SBP	Yes	Yes	Yes	Tested
AT2G03710	SEP4	MADS	Yes	Yes	Yes	Tested
AT2G26580	YAB5	C2C2-YABBY	Yes	Yes	Yes	Tested
AT2G33860	ETT	ARF	Yes	Yes	Yes	Tested
AT2G38880	NF-YB1	CCAAT	Yes	Yes	Yes	Tested
AT2G41690	HSFB3	HSF	Yes	Yes	Yes	Tested
AT2G42400	VOZ2	VOZ	Yes	Yes	Yes	Tested
AT3G06740	GATA15	C2C2-GATA	Yes	Yes	Yes	Tested
AT3G07340	CIB3	bHLH	Yes	Yes	Yes	Inferred
AT3G16870	GATA17	C2C2-GATA	Yes	Yes	Yes	Both
AT3G20910	NF-YA9	CCAAT	Yes	Yes	Yes	Tested
AT3G24050	GATA1	C2C2-GATA	Yes	Yes	Yes	Tested
AT3G25730	EDF3	AP2-EREBP	Yes	Yes	Yes	Tested
AT3G27010	TCP20	TCP	Yes	Yes	Yes	Tested
AT3G28910	MYB30	MYB	Yes	Yes	Yes	Both
AT3G30260	AGL79	MADS	Yes	Yes	Yes	Inferred
AT3G50870	HAN	C2C2-GATA	Yes	Yes	Yes	Tested
AT3G51080	GATA6	C2C2-GATA	Yes	Yes	Yes	Tested
AT3G60390	HAT3	HB	Yes	Yes	Yes	Tested
AT3G60530	GATA4	C2C2-GATA	Yes	Yes	Yes	Tested
AT4G02670	IDD12	C2H2	Yes	Yes	Yes	Inferred
AT4G08150	KNAT1/BP	HB	Yes	Yes	Yes	Both
AT4G11070	WRKY41	WRKY	Yes	Yes	Yes	Inferred
AT4G16780	HAT4	HB	Yes	Yes	Yes	Both
AT4G28790	bHLH23	bHLH	Yes	Yes	Yes	Inferred
AT4G36900	DEAR4	AP2-EREBP	Yes	Yes	Yes	Both
AT4G40060	ATHB16	HB	Yes	Yes	Yes	Tested
AT5G02840	RVE4	MYB-related	Yes	Yes	Yes	Both
AT5G04340	ZAT6	C2H2	Yes	Yes	Yes	Both
AT5G37020	ARF8	ARF	Yes	Yes	Yes	Tested
AT5G50915		bHLH	Yes	Yes	Yes	Inferred
AT5G60910	FUL	MADS	Yes	Yes	Yes	Inferred
AT5G63790	NAC102	NAC	Yes	Yes	Yes	Inferred
AT5G65310	ATHB5	HB	Yes	Yes	Yes	Tested
AT1G02220	NAC003	NAC	Yes	No		no motif

AT1G13880	ELM2	MYB-related	No			
AT1G14685	BPC2	BBR/BPC	Yes	No		no motif
AT1G16490	MYB58	MYB	No			Tested
AT1G20900	ESC	AT-Hook	No			
AT1G46408	AGL97	MADS	No			
AT1G65360	AGL23	MADS	No			
AT1G68480	JAG	C2H2	No			no motif
AT1G69580		G2-like	No			
AT1G75710		C2H2	No			
AT1G76510		ARID	No			
AT2G14760		bHLH	No			
AT2G16770	BZIP23	bZIP	No			
AT2G17770	BZIP27	bZIP	No			
AT2G24840	AGL61	MADS	Yes	No		no motif
AT2G28540		unknown	No			
AT2G31380	STH	Orphans	Yes	No		no motif
AT2G35270	GIK	AT-Hook	No			
AT2G35430		C3H	Yes	No		no motif
AT2G37060	NF-YB8	CCAAT	No			
AT2G39880	MYB25	MYB	No			
AT2G42300		bHLH	Yes	No		Inferred
AT2G45160	HAM	GRAS	Yes	No		no motif
AT3G01530	MYB57	MYB	Yes	No		Tested
AT3G08500	MYB83	MYB	No			Tested
AT3G11100	VFP3	Trihelix	No			
AT3G16500	PAP1	AUX/IAA	No			
AT3G18650	AGL103	MADS	No			
AT3G19360		C3H	Yes	No		no motif
AT3G20640		CCAAT	No			
AT3G24140	FAMA	bHLH	Yes	No		no motif
AT3G25710	TMO5	bHLH	Yes	No		no motif
AT3G26640	LWD2	WD40	No			
AT3G45260	BIB	C2H2	No			Tested
AT3G55560	AHL15	AT-Hook	No			
AT3G57180	BPG2	unknown	No			
AT3G58630		Trihelix	No			Tested
AT3G58680	MBF1B	MBF1	No			
AT3G61950	bHLH67	bHLH	Yes	No		no motif
AT3G61970	NGA2	ABI3VP1	Yes	No		no motif
AT4G24440		C2C2-GATA	No			
AT4G28190	ULT1	ULT	No			
AT4G30180		unknown	No			
AT4G31420	REIL1	C2H2	No			
AT4G35700	DAZ3	C2H2	No			
AT4G36740	ATHB40	HB	Yes	No		Tested
AT4G38960	BBX19	Orphans	No			
AT5G01200		MYB	No			
AT5G02030	RPL	HB	Yes	No		no motif
AT5G05830		PHD	No			
AT5G06160	ATO	C2H2	No			

AT5G08190	NF-YB12	CCAAT	No			
AT5G09460	SACL1	bHLH	No			
AT5G09780	REM25	ABI3VP1	Yes	No		no motif
AT5G11270	OCP3	HD	No			
AT5G14000	NAC084	NAC	No			
AT5G18090		ABI3VP1	Yes	No		Tested
AT5G22290	ANAC089	NAC	Yes	No		no motif
AT5G25890	IAA28	AUX/IAA	No			
AT5G39750	AGL81	MADS	No			
AT5G45580		G2-like	No			Tested
AT5G49420		MADS	No			
AT5G49490	AGL83	MADS	No			
AT5G49700	AHL17	AT-Hook	No			
AT5G53660	GRF7	GRF	Yes	No		no motif
AT5G56840		MYB-related	No			Tested
AT5G56900		C3H	No			
AT5G57660	COL5	C2C2-CO-like	Yes	No		no motif
AT5G61470		C2H2	No			
AT1G01030	NGA3	ABI3VP1	Yes	Yes	No	Inferred
AT1G08010	GATA11	C2C2-GATA	Yes	Yes	No	Tested
AT1G12630		AP2-EREBP	Yes	Yes	No	Both
AT1G53170	ERF8	AP2-EREBP	Yes	Yes	No	Tested
AT1G54060	ASIL1	Trihelix	Yes	Yes	No	Tested
AT1G68800	TCP12	TCP	Yes	Yes	No	Inferred
AT1G69010	BIM2	bHLH	Yes	Yes	No	Tested
AT1G72360	ERF73	AP2-EREBP	Yes	Yes	No	Both
AT2G18380	HANL1	C2C2-GATA	Yes	Yes	No	Tested
AT2G40220	ABI4	AP2-EREBP	Yes	Yes	No	Both
AT2G40970	MYBC1	G2-like	Yes	Yes	No	Inferred
AT2G41940	ZFP8	C2H2	Yes	Yes	No	Tested
AT3G23240	ERF1	AP2-EREBP	Yes	Yes	No	Tested
AT3G61630	CRF6	AP2-EREBP	Yes	Yes	No	Inferred
AT4G13620		AP2-EREBP	Yes	Yes	No	Inferred
AT4G28140		AP2-EREBP	Yes	Yes	No	Both
AT4G32040	KNAT5	HB	Yes	Yes	No	Inferred
AT5G13790	AGL15	MADS	Yes	Yes	No	Tested
AT5G13910	LEP	AP2-EREBP	Yes	Yes	No	Both
AT5G52020		AP2-EREBP	Yes	Yes	No	Tested
AT5G61890	ERF114	AP2-EREBP	Yes	Yes	No	Inferred
AT1G76710	ASHH1	Methyltransf	No			
AT2G20760	CLC1	Clathrin light	No			
AT2G30410	KIESEL	Tubulin bind	No			
AT3G05155		Major facilita	No			
AT4G30860	ASHR3	trxG	No			
AT4G33540		Metallo b-lac	No			
AT5G63730	ARI14	E3 Ligase	No			

Table S4: Y1H results

Library 2 (de Folter library)

Locus	Gene	st growth ma
AT1G01030	NGA3	Trp
AT2G33860	ETT	Trp
AT2G35270	GIK	Trp
AT3G61970	NGA2	Trp
AT4G08150	BP	Trp
AT4G36930	SPT	Trp
AT5G02030	RPL	Trp
AT5G60450	ARF4	Trp

Library 3 (own library)

Locus	Gene	st growth ma
AT1G08465	YAB2	Leu
AT1G13400	NUB	Leu
AT1G23420	INO	Leu
AT1G24260	Sep-03	Leu
AT1G30330	ARF6	Leu
AT1G30490	PHV	Leu
AT1G43850	SEU	Leu
AT1G52150	CNA	Leu
AT1G69180	CRC	Leu
AT2G03710	Sep-04	Leu
AT2G26580	YAB5	Leu
AT2G34710	PHB	Leu
AT3G47730	ATH1	Leu
AT4G00180	YAB3	Leu
AT4G01500	NGA4	Leu
AT4G18960	AG	Leu
AT4G25520	SLK1	Leu
AT4G28190	ULT1	Leu
AT4G32551	LUG	Leu
AT5G60690	REV	Leu
AT5G62090	SLK2	Leu
